# Draft Genome Sequences of Three Enterococcus casseliflavus Strains Isolated from the Urine of Healthy Bovine Heifers (Gyr Breed)

**DOI:** 10.1128/MRA.00386-20

**Published:** 2020-05-21

**Authors:** Silvia Giannattasio-Ferraz, Laura Maskeri, André P. Oliveira, Edel F. Barbosa-Stancioli, Catherine Putonti

**Affiliations:** a Departamento de Microbiologia, Instituto de Ciências Biológicas, Universidade Federal Minas Gerais, Belo Horizonte, MG, Brazil; b Bioinformatics Program, Loyola University Chicago, Chicago, Illinois, USA; c Empresa de Pesquisa Agropecuária de Minas Gerais, Uberaba, MG, Brazil; d Department of Biology, Loyola University Chicago, Chicago, Illinois, USA; e Department of Computer Science, Loyola University Chicago, Chicago, Illinois, USA; f Department of Microbiology and Immunology, Stritch School of Medicine, Loyola University Chicago, Maywood, Illinois, USA; Broad Institute

## Abstract

Enterococcus casseliflavus is a commensal bacterium present in the intestinal microbiota of different animals. Previous studies have found that strains isolated from livestock are often resistant to many different antibiotics. Here, we present three E. casseliflavus strains, UFMG-H7, UFMG-H8, and UFMG-H9, isolated from urine collected from healthy dairy heifers in Brazil.

## ANNOUNCEMENT

Enterococcus casseliflavus is a commensal of the intestinal tract of livestock animals, such as cattle, horses, and sheep (1, 2). Antibiotic resistance is prevalent within this species, with isolates from livestock animals being reported as resistant to vancomycin, tetracycline, erythromycin, ampicillin, and gentamicin (3–5). Here, we report the draft genome sequences of three strains of E. casseliflavus, strains UFMG-H7, UFMG-H8, and UFMG-H9, which were isolated from urine collected from healthy dairy cattle in Brazil.

Sample collection took place at the Agricultural Research Company of Minas Gerais State in May 2019 and was previously approved by the Ethics Committee in Animal Experimentation of the Universidade Federal de Minas Gerais, Brazil (CEUA/UFMG approval number 40/2019). All of the assays performed were in accordance with relevant guidelines. The heifers sampled were from a herd composed of pure-by-origin Gyr cattle. For sampling, the vulva of the heifer was washed with distilled water and soap. Midstream urine was collected using a 50-ml sterile tube. The material was kept at −20°C for no more than 48 h, when it was brought back to the laboratory for processing. Aliquots of 2 ml were centrifuged, and the supernatant was plated on lysogeny broth (LB) agar plates. Plates were incubated overnight at 37°C, and then single colonies were grown in LB medium overnight at 37°C. This process of plating and liquid culture growth was repeated three times until pure colonies were obtained. A single colony was inoculated in LB liquid medium and grown overnight at 37°C under agitation. The DNA was extracted using the Qiagen DNeasy UltraClean microbial kit (Qiagen, Hilden, Germany) and quantified using a Qubit fluorometer. The 16S rRNA gene sequences were amplified using the primers 63f and 1387r (6) and sequenced by GENEWIZ (South Plainfield, NJ, USA) via Sanger sequencing using each primer. The 16S rRNA gene sequences produced were then queried against the NCBI 16S rRNA sequence database to ascertain the species of the isolates. Purified DNA was subjected to whole-genome sequencing at the Microbial Genomic Sequencing Center (MiGS) at the University of Pittsburgh. DNA libraries were prepared by the MiGS as follows: the DNA was fragmented using a tagmentation enzyme (Illumina), and indices were attached by PCR. Genome sequencing was conducted using the NextSeq 550 platform. Sequencing produced 2 × 150-bp reads. These reads were trimmed using Sickle v1.33 (https://github.com/najoshi/sickle) and assembled using SPAdes v3.13.0 with the only-assembler option for k values of 55, 77, 99, and 127 (7). After assembly, the genome coverage was calculated using BBMap v38.47 (https://sourceforge.net/projects/bbmap), and the genomes were annotated by the NCBI Prokaryotic Genome Annotation Pipeline (PGAP) v4.11 (8). The genomes were examined for plasmids using PlasmidFinder v2.0 (9) and for antibiotic resistance genes using ResFinder v3.2 (10). Default parameters were used for all software tools unless otherwise noted.

Table 1 lists the genome statistics for the three E. casseliflavus bovine urinary isolates. Both UFMG-H7 and UFMG-H8 were found to contain an Inc18 plasmid (67.7 kbp and 36.7 kbp long, respectively). UFMG-H9 was not found to contain a plasmid. All three strains were found to contain vancomycin resistance genes. These genes were not carried by plasmids; rather, they were contained in the chromosome. No other antibiotic resistance genes were detected. Further analyses for better understanding of the circulation and role of these bacteria within the urinary tract are relevant to improve our knowledge regarding the bovine microbiota.

**TABLE 1:** Genome statistics for E. casseliflavus strains

Parameter	Data for strain:		
	UFMG-H7	UFMG-H8	UFMG-H9
Length (bp)	3,545,372	3,635,178	3,622,925
No. of contigs	19	26	26
Genome coverage (×)	47	111	50
*N*_50_ (bp)	282,784	503,206	447,071
No. of raw read pairs	1,423,449	1,464,825	1,405,112
GC content (%)	45.32	41.03	45.69
No. of coding genes	3,238	3,347	3,315
No. of tRNAs	53	53	53
No. of plasmids	1	1	0

### Data availability.

These whole-genome sequencing projects are available in GenBank under the accession numbers JAAVMT000000000 (UFMG-H7), JAAVMP000000000 (UFMG-H8), and JAAVMR000000000 (UFMG-H9). Raw data are available under SRA accession numbers SRR11455848 (UFMG-H7), SRR11455647 (UFMG-H8), and SRR11455846 (UFM-H9). These genome sequences are part of BioProject accession number PRJNA615899.
